# Effect of Pink Perch Gelatin on Physiochemical, Textural, Sensory, and Storage Characteristics of Ready-to-Cook Low-Fat Chicken Meatballs

**DOI:** 10.3390/foods12050995

**Published:** 2023-02-26

**Authors:** Khushboo, Nutan Kaushik, Kristina Norne Widell, Rasa Slizyte, Asha Kumari

**Affiliations:** 1 Amity Food and Agriculture Foundation, Amity University Uttar Pradesh, Noida 201313, India; 2 Amity Institute of Food Technology, Amity University Uttar Pradesh, Noida 201313, India; 3 Department of Fisheries and New Biomarine Industry, SINTEF Ocean, Brattørkaia 17C, 7010 Trondheim, Norway

**Keywords:** meatballs, pink perch gelatin, low-fat, textural profile analysis, hedonic analysis, storage study

## Abstract

In recent years consumer demand for low-fat convenience food is increasing rapidly. This study was designed to develop low-fat ready-to-cook (RTC) chicken meatballs using pink perch gelatin. Meatballs were prepared using different concentrations of fish gelatin (3%, 4%, 5%, and 6%). The effect of fish gelatin content on the physico-chemical, textural, cooking, and sensory properties of meatballs was studied. Further, the shelf-life of meatballs was also studied at 4 °C for 15 days and −18 °C for 60 days. The addition of fish gelatin to meatballs decreased the fat content by 67.2% and 79.7% and increased the protein content by 20.1% and 66.4% in comparison to control and Branded Meatballs, respectively. As compared to the Control Meatballs, the addition of fish gelatin also reduced hardness by 26.4% and increased yield and moisture retention in the RTC meatballs by 15.4% and 20.9%, respectively. Sensory analysis suggested that a 5% fish gelatin addition in meatballs has the best acceptability among all tested treatments. Storage study indicated that the addition of fish gelatin to RTC meatballs delayed lipid oxidation during both refrigerated and frozen storage. The results suggested that pink perch gelatin can be used as a fat replacer in chicken meatballs and can potentially increase their shelf-life.

## 1. Introduction

The fat component in the food matrix plays an important role in nutrition and also contributes immensely towards organoleptic properties. However, excessive consumption of fat, especially cholesterol and saturated fats, is associated with adverse health conditions and chronic diseases such as type 2 diabetes mellitus, obesity, cardiovascular diseases, and atherosclerosis [1,2]. Therefore, various health organizations are promoting reduced consumption of fat food products. In addition, due to increased consumer awareness regarding the health ramifications of fat-rich food, a shift towards the consumption of low-fat food is being observed in the global market.

Fat plays a significant role in ground meat products such as meatballs. Meatballs are a mixture of an emulsion of ground meat, fat, water, and other ingredients such as flour and spices. It has a relatively high amount of fat, which stabilizes the meat emulsion and elevates organoleptic properties such as texture and flavor [2]. The decrease in palatability associated with fat reduction is the major concern regarding the acceptability of low-fat meat products [3]. In addition, a lowering of the fat content has also been reported to cause undesirable changes in the textural attributes of meat products such as increased firmness [4].

Important properties required for the development of low-fat meatballs are emulsion stability, water binding capacity, melt-in-mouth property, and textural modification. In order to address these issues and improve the nutritional properties of low-fat meat products, fat replacers are being used. On the basis of composition, fat replacers can be classified into three categories: carbohydrates, proteins, and lipid modification-based fat replacers. Protein-based fat replacer, however, provides an added advantage of increasing the nutritional quality of meat products by augmenting the overall protein content of the product. In addition, dietary protein has been observed to be the most satiating macronutrient due to its ability to modify metabolites (e.g., amino acid) and hormones (e.g., insulin and gastrointestinal hormones) [5]. They are also considered to influence metabolic targets for both weight loss and weight management [6]. Gelatin is one of the protein-based fat replacers which can be used in the production of low-fat meatballs.

Gelatin not only provides nutritional and textural benefits, but also imparts antioxidant activity due to its DPPH radical scavenging activity [7]. It is also reported to reduce shrinkage and improve cooking yields in meat products such as meat sausage [8]. The addition of gelatin in meat products improves their functional and textural properties by the virtue of two mechanisms: the first is gelling behavior, i.e., the ability to form gel structure that improves texture and water binding capacity, and the second is surface behavior, i.e., the ability to increase the emulsification, foaming, cohesion, and adhesion which stabilize the colloidal systems [9]. Previous studies have reported the use of fish gelatin extracted from different species such as bighead carp, grass carp, and tilapia to develop low-fat mayonnaise, low-fat milk cream, and low-fat yoghurt, respectively [10,11,12]. However, no study has been reported on the use of pink perch gelatin to develop low-fat chicken meatballs. Recently, pink perch is gaining commercial attention due to its strong gelling capacity, which makes it ideal for gelatin extraction [13]. Gelatin extracted from pink perch is reported to have high gel strength, good water-holding capacity, and emulsion activity [14,15]. In addition, the pink perch gelatin has a melting temperature close to normal human body temperature, which provides the melt-in-mouth property, thus imparting fat-like properties to low-fat products. Moreover, the addition of fish gelatin to meatballs is conducive to ecological sustainability and food security, as most of the fish skin and bones used as raw material for the production of fish gelatin are conventionally being discarded by the fish processing industry which causes environmental pollution [16]. As per our knowledge, this is the first study to report the use of pink perch gelatin as a fat replacer for the development of low-fat ready-to-cook chicken meatballs.

Other ingredients such as meat binders and starch present in meatballs are also known to moderate the functional characteristics such as water-holding capacity (WHC), texture, palatability, and appearance [17,18]. Black gram flour is a common ingredient used in meat products [19,20]. It is added to increase fat emulsification, water retention, formation of meat structure, and to reduce cost [21,22]. Black gram flour forms complex gel networks with meat proteins, which can trap water and other compounds thus forming stronger bonds between them. This phenomenon helps to achieve a higher water retention in the meat matrix during processing.

The objective of this research was to develop low-fat ready-to-cook (RTC) chicken meatballs using pink perch gelatin and to determine the efficacy of pink perch gelatin in improving the physico-chemical characteristics, functional properties, and sensory acceptance of the RTC meatballs as compared to control and Branded Meatballs. Further, the storage stability of low-fat RTC meatballs under refrigerated (4 °C for 15 days) and frozen conditions (−18 °C for 60 days) was also studied.

## 2. Materials and Methods

### 2.1. Materials

Chicken mincemeat was procured from a local market. Potato starch, hydrogenated fat, and a spice mix ((garam masala: a mixture of spices containing clove, cinnamon, cardamom, black pepper, mace, and nutmeg), cumin powder, turmeric powder, red chili powder, garlic powder, ginger powder, and common salt) were procured locally. Roasted black gram flour was prepared as per the method of Modi et al. [23]. Branded Meatballs were procured from a local market based on preliminary screening, popularity, and availability. The composition of the Branded Meatballs as claimed on the packaging was chicken meat (56.16%), soya (10%), water, edible vegetable oil, batter (wheat flour, corn flour, corn starch, salt, and emulsifier sodium carboxymethyl cellulose (INS 466)), chili, onion, garlic, ginger, coriander, iodized salt, spices and condiments, and sodium nitrite (INS 250).

### 2.2. Chemicals

Glacial acetic acid and sodium hydroxide were procured from Thermo Fischer Scientific, New Delhi, India. Thiobarbituric acid was procured from LOBA (Loba Chemie Pvt. Ltd., Mumbai, India). All the chemicals used for evaluating the quality of the product were of AR grade.

### 2.3. Pink Perch Gelatin Preparation

Pink perch gelatin was prepared as per the method of K. et al. [14]. Pink perch skin and bones were cut into small pieces. The pieces were mixed with distilled water in a ratio of 1:3 (*w*/*v*) and the extraction was done at pH 3, 75 °C for 30 min. The pH of the aqueous solution was adjusted using glacial acetic acid. The reaction mixture was neutralized to pH (6–6.5) after the reaction using 4M NaOH. Subsequently, the non-solubilized material was filtered. The filtrate containing solubilized gelatin was freeze-dried using a lyophilizer (SNS FD-50, S N Solutions, Noida, India).

### 2.4. Preparation of Ready-to-Cook Chicken Meatballs

Chicken mince was divided into five equal parts, one each for control, 3%, 4%, 5%, and 6% gelatin treatment. The control sample was prepared without fish gelatin and had 4.5% vegetable oil while other treatment groups were prepared by incorporation of fish gelatin in varying concentrations (3–6%) without the addition of any vegetable oil (Table 1). All the experimental batches had 8% roasted black gram flour as per the method of Modi et al. [23]. All the ingredients were mixed manually to obtain a meatball dough and meatballs (20 g each) were formed. Ready-to-cook meatball samples were stored in metalized polyester pouches and stored at 4 °C for 15 days and −18 °C for 60 days for the shelf-life studies. For sensory and other physico-chemical analysis, frozen meatballs were fried at 145 ± 5 °C for 5 min in 500 mL refined sunflower oil.

### 2.5. Physico-Chemical Characterization of Meatballs

#### 2.5.1. Proximate Analysis

Moisture, fat, and ash content of raw and fried meatballs were estimated as per the AOAC methods 950.46, 985.15, and 920.153, respectively [24]. Protein content was estimated by Dumas’ method of protein estimation using Rapid MAX N Exceed nitrogen combustion analyzer (Elementar India Pvt. Ltd., Haryana, India) [25]. The sample (50 mg) was weighed in a steel crucible and the crucible was placed in a combustion chamber. Aspartic acid (≥98%, Sigma Aldrich chemicals Pvt. Ltd., Bangalore, India) was used as reference material to calibrate the nitrogen analyzer. The nitrogen-to-protein conversion factor used for the meatballs was 6.25 [24].

#### 2.5.2. Texture Profile Analysis (TPA)

The textural profile analysis of raw and fried meatballs was done for determining the parameters such as hardness (N), cohesiveness, springiness (cm), gumminess (N), and chewiness (N.cm) using a TA-XT Plus texture analyzer (Stable Micro Systems, Surrey, UK). A 36 mm cylindrical probe was attached to a 50 kg load cell to compress the sample to 75% of its original height twice in two cycles at a test speed of 1 mm/s.

#### 2.5.3. 2,2-Diphenylpicrylhydrazyl (DPPH) Radical Scavenging Activity

DPPH radical scavenging activity of the sample was determined as per the method of Wojtasik-Kalinowska et al. [26]. A sample (2.5 g) from each treatment group of meatballs was crushed manually and homogenized in 7.5 mL of ethanol for 10 min using a vortex. The homogenized samples were centrifuged for 15 min at 6000× *g* at room temperature (25 °C). The supernatant (0.5 mL) was added to 3.5 mL of 0.1 mM ethanolic DPPH and mixed thoroughly for 30 s. The prepared solution was stored in the dark at room temperature for 30 min. Absorbance at 517 nm was measured using UV-VIS spectrophotometer (LMSPU1000B, Labman Scientific Instruments Pvt. Ltd., Chennai, India). The ethanol solution without the sample was taken as blank. The DPPH radical scavenging activity was calculated using Equation (1).

(1) $$\mathrm{DPPH}\;\mathrm{radical}\;\mathrm{scavenging}\;\mathrm{activity}\;=1-\frac{A_{\mathrm{Sample}}}{A_{\mathrm{Blank}}}\times 100$$

where, A_sample_—Absorbance of the DPPH solution with the tested sample; A_blank_—Absorbance of the DPPH solution with 99.5% ethanol.

### 2.6. Determination of Cooking Parameters of Meatballs

#### 2.6.1. Cooking Yield

The cooking yield of meatballs was determined by measuring the weight of each meatball before and after cooking. The yield was calculated as per Equation (2).

(2) $$\mathrm{Cooking}\;\mathrm{yield}\;\left(\%\right)=\frac{\mathrm{Weight}\;\mathrm{of}\;\mathrm{fried}\;\mathrm{meatball}}{\mathrm{Weight}\;\mathrm{of}\;\mathrm{raw}\;\mathrm{meatball}}\times 100$$

#### 2.6.2. Moisture Retention

Moisture retention value is the measure of the amount of moisture retained in the fried meatballs. It was calculated as per Equation (3) described by Serdaroglu et al. [19].

(3) $$\mathrm{Moisture}\;\mathrm{Retention}\;\left(\%\right)=\frac{\mathrm{Percent}\;\mathrm{yield}\;\mathrm{of}\;\mathrm{meatball}\;\times \;\mathrm{Percent}\;\mathrm{moisture}\;\mathrm{in}\;\mathrm{fried}\;\mathrm{meatball}}{100}$$

#### 2.6.3. Shrinkage

Shrinkage in the meatballs was determined by measuring the diameter of the meatballs before and after frying in the oil using a vernier caliper [19]. The shrinkage was measured as per Equation (4).

(4) $$\mathrm{Shrinkage}\;\left(\%\right)=\frac{\mathrm{Diameter}\;\mathrm{of}\;\mathrm{raw}\;\mathrm{meatball}-\mathrm{Diameter}\;\mathrm{of}\;\mathrm{fried}\;\mathrm{meatball}}{\mathrm{Diameter}\;\mathrm{of}\;\mathrm{raw}\;\mathrm{meatball}}\times 100$$

### 2.7. Sensory Evaluation

Sensory evaluation of the meatballs was done using a 9-point hedonic scale (9–extremely desirable and 1–extremely undesirable). The evaluation was done by a panel of 30 semi-trained members (15 males and 15 females; age range: 24–45 years) from Amity University Uttar Pradesh, Noida, India. All the panelists had prior experience in meat product assessment. Fried meatballs were served warm to the semi-trained panelists. Samples were presented with codes in random order. The experiment was done in a well-lit room and water was provided as a palate cleanser between samples. Meatballs were ranked for appearance, taste, smell, texture, and overall acceptability.

### 2.8. Shelf-Life Studies

The effects of gelatin addition on the shelf-life of RTC meatballs were studied during refrigerated storage conditions (4 °C for 15 days) and frozen storage conditions (−18 °C for 60 days) and compared with Control and Branded Meatballs. At refrigerated storage conditions the samples were drawn at intervals of 0, 3, 6, 9, 12, and 15 days and at frozen storage conditions the samples were drawn at intervals of 0, 15, 30, 45, and 60 days for analysis. The meatball samples were analyzed for their physico-chemical (water holding capacity, thiobarbituric acid value, free fatty acid value, pH, and color) and microbiological parameters.

#### 2.8.1. Water Holding Capacity (WHC)

WHC of meatballs was measured as per the method of Bouton et al. [27]. The meatball (5 g) was centrifuged at 9000× *g* rpm for 30 min at 4 °C and weight was measured before and after centrifugation. The water holding capacity was calculated using Equation (5).

(5) $$\mathrm{Water}\;\mathrm{Holding}\;\mathrm{Capacity}\;\left(\mathrm{WHC}\right)\;\%\;=1-\frac{T}{M}\times 100$$

where,

T = Difference between A and B (i.e., B—A)
B is the weight of the sample before frying
A is the weight of the sample after frying and centrifuging
M is the total water content in the sample

#### 2.8.2. Thiobarbituric Acid Value (TBARS)

The thiobarbituric acid value of the meatballs was determined as per the method of Schmedes et al. [28]. The meatball sample (5 g) was mixed with 10 mL of 20% TCA for 30 s using a vortex. The mixture was filtered. The filtrate (2 mL) was added to 2 mL of 0.02 M aqueous thiobarbituric acid solution. The contents were incubated for 100 °C for 30 min and further cooled under tap water. Absorbance was measured at 532 nm using UV-VIS spectrophotometer and TBA value was calculated using malondialdehyde as the standard (expressed as mg MDA per kg).

#### 2.8.3. Free Fatty Acid Percentage (FFA)

The free fatty acid percentage of meatballs was estimated as per the method of Bienkiewicz et al. [29]. The meatball sample (2 g) was mixed with 30 mL of chloroform and homogenized at 6000× *g* rpm for 1 min. The mixture was filtered to remove solid particles. A few drops of 1% ethanolic phenolphthalein indicator were added to the filtrate. The filtrate was titrated against 0.01N potassium hydroxide. Free fatty acid was expressed as % oleic acid. The free fatty acid percentage was calculated as per Equation (6).

(6) $$\mathrm{FFAs}\;\mathrm{oleic}\;\mathrm{acid}\;\left(\%\right)=\frac{\left(V_{i}-{\;V}_{f}\right)\times 28.2}{W}$$

V_i_: Initial titrant volume (mL)
V_f_: Final titrant volume (mL)
W: The amount of sample (g)
Conversion factor: 28.2

#### 2.8.4. pH

The Meatball sample (2 g) was homogenized with 20 mL of distilled water for 1 min at room temperature (25 °C) using a vortex. The mixture was used to measure the pH with a digital pH meter (Labman Scientific Instruments Pvt. Ltd., Chennai, India).

#### 2.8.5. Color Analysis

The color of the meatballs was measured using CIEL*a*b* system-based NS810 portable spectrophotometer (Shenzhen Threenh Technology Co. Ltd., Shenzhen, China), calibrated against white and black standards provided with the instrument. The color was measured on three samples at three different locations. The results were expressed in terms of L* (lightness), a* (redness/greenness), and b* (yellowness/blueness).

#### 2.8.6. Microbial Assessment

The homogenized sample (1 g) was dissolved in 10 mL peptone water. Serial dilutions were prepared from 10^−^^1^ to 10^−^^4^ dilution. After that 100 µL of the sample from dilutions was plated on different agar plates by spread plate techniques. Media used for total plate count, yeast and mold, *Salmonella,* and *E. coli* count were nutrient agar, Czapek Dox agar, XLD agar, and EMB agar, respectively. Plates were incubated for 24 h at 37 °C for total plate count, *Salmonella,* and *E. coli* and at 28 °C for 3 days for yeast and mold. The results were expressed as colony forming unit/gram (CFU/g).

### 2.9. Statistical Analysis

All experiments were done in triplicates. Data were analyzed for mean and standard deviation using Microsoft office excel 2016. Data were compared for analysis of variance using IBM SPSS (Version 26.0). The mean value was further compared using the Duncan Multiple Range test. The significance level in the study was *p* < 0.05.

## 3. Result and Discussion

### 3.1. Proximate Composition

#### 3.1.1. Raw Meatballs

The proximate composition of the raw chicken meatballs varied significantly (*p* < 0.05) when replacing vegetable oil with fish gelatin (Table 2). Fish gelatin-incorporated meatballs had 67.2% lower fat content than the control and 79.7% lower fat content than the Branded Meatballs. However, no significant difference was observed between the fat content of meatballs with different concentrations of fish gelatin. Since fish gelatin is composed of more than 90% protein, the protein content of fish gelatin-incorporated RTC meatballs was also significantly higher than the Control and the Branded Meatballs by 20.1% and 66.4%, respectively (*p* < 0.05). The moisture content of the gelatin-incorporated RTC meatballs (64.5%) was also significantly higher than the Control (62.0%) and Branded Meatballs (62.1%). This could be attributed to the water-holding capacity of fish gelatin [30]. A higher moisture content improves the overall acceptability of the product by increasing juiciness. The ash content of the Control Meatballs was observed to be significantly higher than the gelatin-incorporated and the Branded Meatballs (*p* < 0.05).

#### 3.1.2. Fried Meatballs

Similar to raw meatballs, a significant variation in the proximate composition of fried meatballs was observed with the replacement of vegetable oil with fish gelatin (Table 2). The results suggested that the addition of fish gelatin significantly reduced the fat content of fried meatballs as compared to the fried control sample and branded sample, respectively (*p* < 0.05). The fat content of fried Branded Meatballs was observed to be two times more than the fat content of fried fish gelatin-incorporated meatballs. It was also observed that an increase in fish gelatin concentration led to a decrease in fat absorption in the fried meatballs. This observation could be explained by the fact that during the frying process, gelatin forms a gel matrix that seals the moisture content of meat and prevents further penetration of external fat inside the system. During frying, due to loss of moisture, overall protein concentration in the meatballs increased with respect to raw meatballs. Similar to raw meatballs, the protein content of gelatin-incorporated fried meatballs was also significantly higher than the fried control and the Branded Meatballs (*p* < 0.05). A linear relation was observed between the protein content and the fish gelatin concentration in meatballs.

A decrease in the moisture content of the fried meatballs with respect to raw meatballs was observed in all treatment groups. This was due to moisture removal during the process of frying. The result also suggested a positive correlation between the moisture content and fish gelatin concentration (r = 0.99) in the meatballs. No significant difference in the ash content of fried meatballs was observed with the addition of different concentrations of fish gelatin. Our results were in accordance with Jridi et al. [8] and Pereira et al. [31] who reported alteration in the chemical composition of sausage and frankfurters, respectively, with the addition of gelatin. The results from the proximate analysis suggested that the addition of pink perch gelatin to meatballs can effectively reduce the fat content along with improving the protein and moisture content.

### 3.2. Texture Profile Analysis (TPA)

The textural characteristics of fried meatballs have a significant impact on the consumer preference. Textural properties of meat products are affected by various factors such as product composition, processing technique, and cooking parameters [32]. The frying of the meat product at a high temperature significantly alters the textural properties due to moisture loss, gel matrix development, and surface hardening because of crust formation. The results obtained in this study also indicated that the process of frying has led to modification in the textural parameters of all meatballs irrespective of the treatment. As compared to the raw meatballs, there was a significant increase in hardness, springiness, cohesiveness, gumminess, and chewiness of all meatballs after frying (*p* < 0.05) (Table 3). The textural properties of fried meatballs were also influenced by the addition of fish gelatin (Table 3). The results indicated a decrease in the hardness of the fried meatballs with an increase in the concentration of fish gelatin. This could be due to higher moisture retention with an increase in fish gelatin concentration leading to a softer texture. The hardness of the fried fish gelatin-incorporated meatball was observed to be significantly lower than the fried control sample without fish gelatin but slightly higher than the Branded Meatballs. A similar result has been reported for the textural properties of fish balls prepared with the incorporation of tilapia gelatin [33]. Springiness is representative of the deformation in the system after the removal of external compressive force whereas cohesiveness indicates resistance due to internal bonds. In this study, no significant difference was observed between the springiness of fried meatballs of different treatments, whereas the cohesiveness of fried Branded Meatballs was observed to be slightly higher than fried control and fried fish gelatin-incorporated meatballs. The addition of fish gelatin resulted in a good quality of the meatballs which can easily reform their structure after the removal of compressive forces. Based on the findings, it was observed that the chewiness of fried fish gelatin-incorporated meatballs was significantly lower than the fried Branded and fried Control Meatballs. The presence of crust has been reported to affect the food’s mechanical properties, as well as its texture and acceptability [34]. Therefore, the development of the crust may be related to the hardness and chewiness of deep-fried meatballs. An inverse relation was also observed between the gumminess of the fried meatballs and the gelatin concentration (Table 3).

### 3.3. 2,2-Diphenylpicrylhydrazyl (DPPH) Radical Scavenging Activity

**Figure 1:**
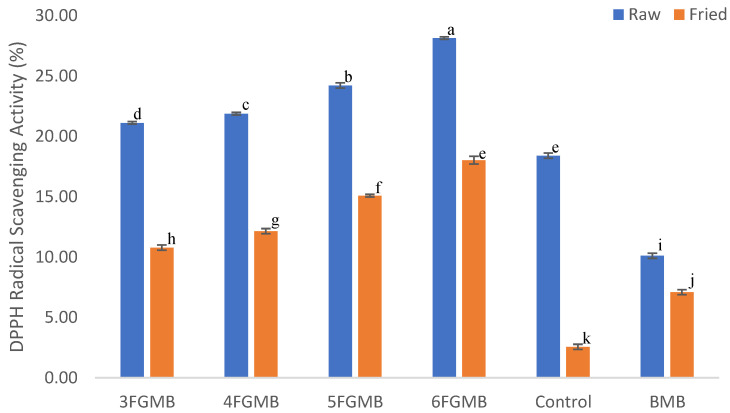
DPPH Radical scavenging activity of raw and fried meatballs. * 3FGMB: 3% Fish Gelatin Meatball, 4FGMB: 4% Fish Gelatin Meatball, 5FGMB: 5% Fish Gelatin Meatball, 6FGMB: 6% Fish Gelatin Meatball, BMB: Branded Meatball, Control: Control Meatball. Error bar indicates the standard deviations from three replications; a–k (↓) different lowercase letters indicate significant difference between different meatballs (*p* < 0.05).

The DPPH scavenging-based antioxidant activity of the fish gelatin-based chicken meatballs ranged from 21.1–28.1% in raw samples and 10.8–18.0% in fried samples (Figure 1). The substitution of vegetable oil with fish gelatin was able to increase the antioxidant activity of the meatballs, therefore controlling oxidation reaction and retarding the development of rancidity. The antioxidant activity of meatballs is by the virtue of gelatin’s ability to quench DPPH free radicals by donating protons [7]. The results indicated a positive correlation (r = 0.99) between fish gelatin concentration and the DPPH scavenging effect in fried meatballs. The highest value of antioxidant activity (28.1%) was observed in 6% fish gelatin-incorporated meatballs. It was observed that all treatment groups of meatballs had a certain amount of antioxidant activity. This could be due to the presence of spices in the control and Fish Gelatin Meatballs whereas in the Branded Meatball the chemical preservative might have imparted a certain amount of antioxidant effect. It was also observed that antioxidant activity of all meatballs, irrespective of the treatment, reduced after frying process. This could be due to antioxidant degradation or usage during the heat treatment. The antioxidant capacity results showed that the pink perch gelatin has a potential to improve the storage and nutritional quality of meatballs.

### 3.4. Cooking Parameters

#### 3.4.1. Cooking Yield

Cooking yield is an important parameter in terms of the commercial value of a product for the industry as it helps in predicting the behavior of the products during frying [35]. The determination of cooking yield is based on the estimation of weight loss incurred by meatballs during frying. It is dependent on the ability of the protein matrix in meatballs to immobilize and retain fat and water during the processing. It was observed in our study that the addition of fish gelatin significantly increased the cooking yield of meatballs in comparison to the Control Meatballs (Table 4). The highest cooking yield of fish gelatin-incorporated meatballs was recorded at 6% fish gelatin concentration (85.6%), which was also observed to be higher than the Branded Meatball (83.8%). An increase in fish gelatin concentration was observed to have a positive correlation (r = 0.96) with meatball yield. Fish gelatin causes water and oil retention which helps in reducing weight loss in meatballs during frying. Therefore, the difference in moisture retention has led to variations in meatball yield. Results were in agreement with the observation of Jirdi et al. [8], who reported a proportional relationship between added gelatin concentration and cooking yield in meat sausage.

#### 3.4.2. Moisture Retention

The results suggested that the meatballs incorporated with fish gelatin had a higher moisture retention capacity as compared to the Control Meatballs and Branded Meatballs (Table 4). This could be explained by the ability of gelatin to covalently form matrices that swell in an aqueous solution, which results in the formation of a gelatin network and hence, increases the moisture retention capacity of the meatballs [8]. The results also depicted a linear relation (r = 0.99) between moisture retention and fish gelatin concentration of meatballs. In this study, the highest moisture retention capacity was observed in meatballs incorporated with 6% fish gelatin (56.5%), which was also observed to be higher than the Branded Meatballs (51.7%).

#### 3.4.3. Shrinkage

In this study, shrinkage was observed in all treatment groups of meatballs irrespective of the source and formulation (Table 4). The process of frying causes loss of moisture and denaturation of protein, resulting in shrinkage of the meatballs. The results suggested that the meatball incorporated with fish gelatin showed a significantly lower shrinkage in comparison to the Control Meatballs. This could be due to the water holding capacity and moisture retention capacity of fish gelatin, leading to a reduction in moisture loss. An inverse relationship was observed between fish gelatin concentration and shrinkage (r = −0.93). The lowest percentage of shrinkage was observed in meatballs with 6% fish gelatin (2.1%).

### 3.5. Sensory Evaluation

**Figure 2:**
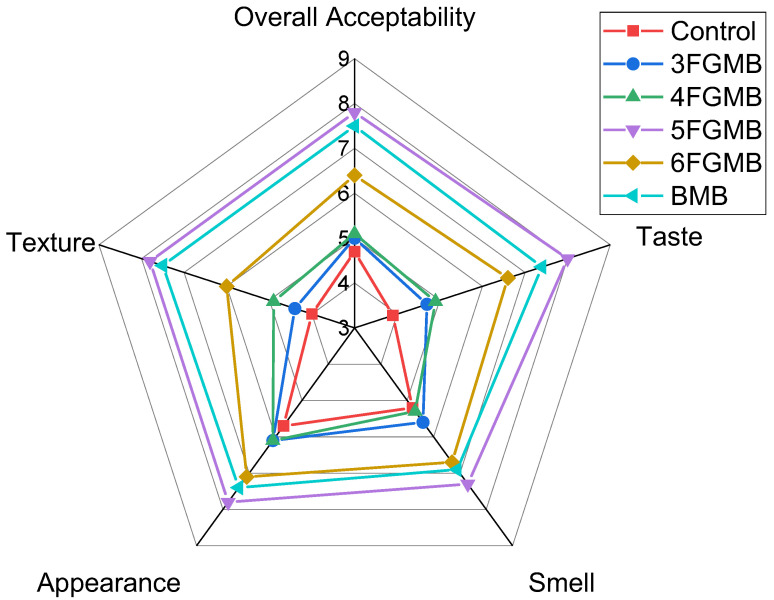
Sensory evaluation scores of meatballs *. * 9—extremely desirable and 1—extremely undesirable; 3FGMB: 3% Fish Gelatin Meatball, 4FGMB: 4% Fish Gelatin Meatball, 5FGMB: 5% Fish Gelatin Meatball, 6FGMB: 6% Fish Gelatin Meatball, BMB: Branded Meatball, Control: Control Meatball.

The sensory evaluation of fried meatballs suggested that the addition of fish gelatin and variation in its concentration had a significant effect on all parameters, i.e., texture, appearance, taste, smell, and overall acceptability (Figure 2). In comparison to other variants of meatballs (fish gelatin-incorporated meatballs and Branded Meatballs), Control Meatballs had the lowest acceptability scores. Meatballs incorporated with fish gelatin had a higher score for appearance in comparison to control and blank meatballs, which could be due to the higher moisture retention. An increase in moisture retention also led to higher retention of the natural juices of meat protein which facilitated the release of flavor compounds. This was also evident from the high taste scores of meatballs formulated with fish gelatin. As per the panelist, no fishy odor was perceptible in fish gelatin-incorporated meatballs. Considering all attributes, meatballs formulated with 5% fish gelatin were most acceptable to panelists. With a further increase in fish gelatin concentration, a decrease in the acceptability of the meatball was observed. This could be due to the fact that as the fish gelatin concentration increased, the chewiness of the meatballs increased as well and they took on a darker color appearance.

### 3.6. Shelf-Life Study

The shelf-life of meat and meat products is majorly influenced by oxidation and microbial contamination, resulting in deterioration of nutritional value, development of unacceptable color, flavor and odor, and toxin production [36]. In this study, on the basis of the highest consumer acceptability during sensory evaluation, a 5% Fish Gelatin Meatball was selected for shelf-life analysis and was compared with the Control Meatball to analyze the effect of gelatin addition on the shelf-life of the meatballs. The shelf-life of Branded Meatball was also determined to analyze the comparability of fish gelatin-incorporated meatballs with respect to commercially available meatball variants. Estimation of shelf-life of 5% Fish Gelatin Meatball, Control Meatball, and Branded Meatball was done at refrigerated (4 °C for 15 days) and frozen temperatures (−18 °C for 60 days) by estimating changes in water holding capacity, thiobarbituric acid value, free fatty acid percentage, pH, color, and microbial count.

#### 3.6.1. Water Holding Capacity (WHC)

**Figure 3:**
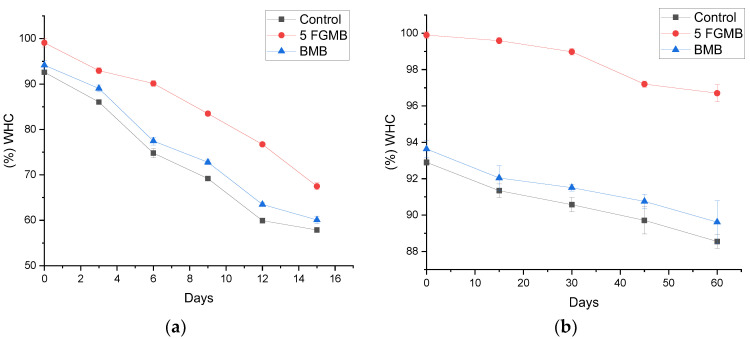
Effect of storage (**a**) at 4 °C for 15 days and (**b**) at −18 °C for 60 days on water holding capacity of meatballs *. *. Control: Control Meatball, 5 FGMB: 5% Fish Gelatin Meatball, BMB: Branded Meatball; Error bar indicates the standard deviations from three replications (*p* < 0.05).

WHC in meat products influence consumer acceptance by positively regulating visual desirability, yield, drip losses, and sensory properties of meatballs. The fish gelatin-incorporated meatball showed a higher WHC in comparison to the Control and the Branded Meatball samples at both storage conditions (*p* < 0.05) (Figure 3a,b). An overall decrease in WHC was observed in all meatballs with the increase in storage time. The rate of decrease in WHC at refrigerated conditions was higher than at frozen conditions. A sharp decrease in WHC at refrigerated conditions after 15 days may be because of the changes in protein structure. Protein denaturation can be a result of an increase in acidity of the system due to enzymatic and microbial spoilage [37]. This results in a reduced ability of meat to hold water molecules. At the frozen condition, the WHC of meatballs is reduced due to the formation of ice-crystals, resulting in myofibrillar shrinkage and partial protein denaturation [38]. Gelatin slows down the ice-crystal formation due to its ability to hold a high amount of water [39], therefore preventing WHC of meatball which was evident in this study. The result suggested gelatin incorporation was effective in improving the WHC of meatballs during both refrigerated and frozen storage conditions.

#### 3.6.2. Thiobarbituric Acid Value

**Figure 4:**
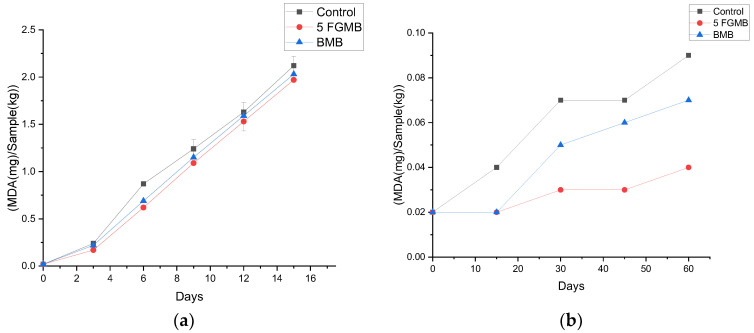
Effect of storage (**a**) at 4 °C for 15 days and (**b**) at −18 °C for 60 days on TBARS value of meatballs *. *. Control: Control meatball, 5 FGMB: 5% Fish Gelatin Meatball, BMB: Branded Meatball. Error bar indicates the standard deviations from three replications (*p* < 0.05).

The oxidative rancidity of lipids is a serious problem that limits the storage stability of meat and meat products. The thiobarbituric acid (TBARS) value is one of the most commonly used measures of lipid oxidation leading to rancidity. From a health and sensory acceptance perspective, a lower value of TBARS is more preferable. During storage at refrigerated conditions, TBARS values of fish gelatin-incorporated meatballs, Control, and Branded Meatballs varied significantly on 6, 9, 12, and 15 days (*p* < 0.05) (Figure 4a). At both frozen and refrigerated storage temperatures, the rate of oxidation in fish gelatin-incorporated meatballs was observed to be lower than in the Control and Branded Meatball samples (Figure 4a,b). This could be due to the antioxidant activity of fish gelatin and its ability to act as a physical barrier between lipid molecules and pro-oxidants present in the system, thus lowering the lipid oxidation rate [40]. A gradual increase in the TBARS values was observed in all treatment groups of meatballs with an increased storage time at both storage conditions. However, the rate of increase in TBARS values of meatballs stored at refrigerated conditions was significantly higher in comparison to the frozen storage conditions (*p* < 0.05). It was also observed that at the end of the storage period the TBA values for all the meatballs were within the acceptable limits at both refrigerated and frozen storage conditions [19]. The results suggested that the fish gelatin was able to effectively retard lipid oxidation at both storage conditions and fish gelatin-incorporated meatballs can retain their oxidative stability at refrigerated conditions for 15 days and at frozen storage temperature for 60 days.

#### 3.6.3. Free Fatty Acid Percentage

**Figure 5:**
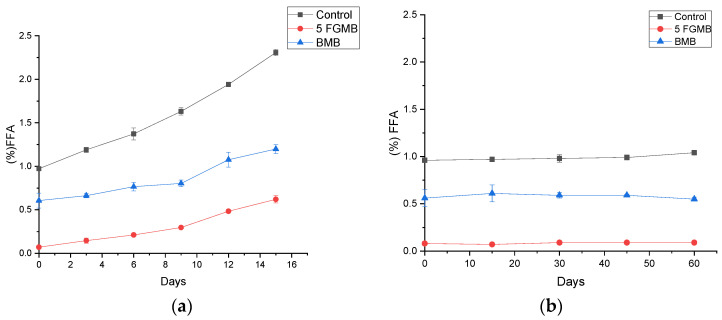
Effect of storage (**a**) at 4 °C for 15 days and (**b**) at −18 °C for 60 days on free fatty acid percentage of meatballs *. *. Control: Control Meatball, 5 FGMB: 5% Fish Gelatin Meatball, BMB: Branded Meatball. Error bar indicates the standard deviations from the three replications (*p* < 0.05).

During storage, meat and meat products undergo enzymatic and microbial degradation due to microorganisms containing lipolytic enzymes resulting in the formation of free fatty acids (FFA) [36]. The estimation of the free fatty acid content helps in determining the storage stability of the product. In this study, an increase in the FFA value with prolonged time was observed in both refrigerated storage and frozen storage. However, the rate of increase of FFA content was significantly higher in the refrigerated storage for 15 days (4 °C) condition than in the frozen storage at 60 days (−18 °C) condition (*p* < 0.05) (Figure 5). A relatively lower rate of increase in FFA content at frozen conditions indicated that frozen storage must be chosen if products are intended to store for more than 2–4 days. The results also suggested that the fish gelatin-incorporated meatballs showed a lower rate of increase in FFA as compared to the Branded and the Control Meatball samples.

#### 3.6.4. pH

**Figure 6:**
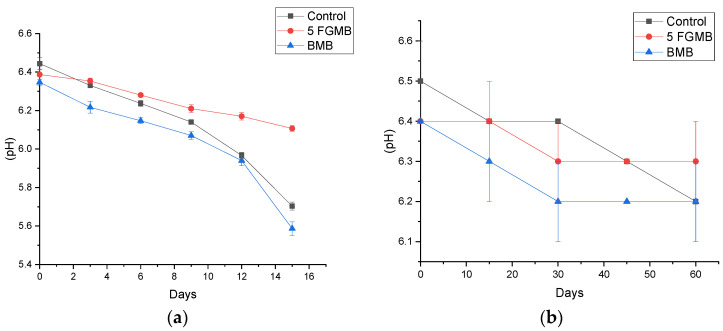
Effect of storage (**a**) at 4 °C for 15 days and (**b**) at −18 °C for 60 days on the pH value of meatballs *. *. Control: Control Meatball, 5 FGMB: 5% Fish Gelatin Meatball, BMB: Branded Meatball. Error bar indicates the standard deviations from three replications (*p* < 0.05).

A decreasing trend in the pH of meatballs was observed at refrigerated storage conditions (4 °C for 15 days) and at frozen (−18 °C for 60 days) storage conditions (Figure 6). The pH value of meatballs stored at 4 °C varied between 6.4 to 5.6 during the storage period of 15 days (Figure 6a). During storage, a decrease in pH may be due to the accumulation of deaminated protein, organic acid, and microbial metabolites formed as a result of enzymatic activity and the growth of microorganisms. Microorganisms metabolize carbohydrates and other compounds present in meat to produce lactic acid and acetic acid which results in a decrease in the pH of the system [41]. The results showed that the decrease in pH of fish gelatin-incorporated meatballs was lower than the Control and Branded Meatballs. This could be due to an increase in the acidity of the meatballs caused by free fatty acid produced as a result of lipid peroxidation during storage. Since the rate of increase of free fatty acid production during refrigerated storage was higher in the Branded and Control Meatballs (Figure 5a), a comparatively rapid decrease in their pH was also observed. Similar results were reported by Rubel et al. [42] in mutton meatballs during refrigerated storage.

pH values of the meatballs stored at −18 °C ranged from 6.5 to 6.2 during the storage period of 60 days (Figure 6b). The results showed that the pH value of the meatballs stored at −18 °C remained relatively stable over the entire 60 days in all treatment groups (*p* < 0.05). In addition, there was no significant difference in pH values (*p* > 0.05) between all combinations of meatballs during the whole storage period, indicating that less quality deterioration can be expected while storing meatballs in frozen condition for up to 60 days.

#### 3.6.5. Color Analysis

The color characteristics influence customer acceptance and preference of the product. A number of factors affect color of the product such as ingredients and their interaction, packaging, processing, and exposure to light [43]. Results indicated that the fish gelatin-incorporated meatballs had a higher lightness value in comparison to the Control Meatballs (Table 5). This could be because of the addition of fish gelatin which was white in color and also due to the swelling of gelatin after getting in contact with water molecules and causing the scattering of light rays. It was also observed that the a* value (redness) of meatballs decreased with the addition of fish gelatin. Jirdi et al. [8] reported similar results regarding the a* value of meat sausages with the addition of cuttlefish gelatin. During storage at refrigerated conditions, a decrease in the lightness value and an increase in the a* value were observed in all treatment groups. This could be due to the browning of the meatballs because of oxidation reactions during the storage period. No significant variation was observed in the color parameters of meatballs during storage at frozen conditions (Table 6).

#### 3.6.6. Microbial Assessment

Total Plate Count (TPC), *Salmonella*, yeast and mold (Y&M), and *E. coli* count of meatballs stored at 4 °C for 15 days and −18 °C for 60 days are shown in Table 7 and Table 8. Microbiological assessment of the product during storage was done to evaluate both the quality and safety of the food product. The maximum permitted microbiological limit set by the Food Safety and Standards Authority of India (FSSAI) for frozen meatballs for Total Plate Count is 1.0 × 10^4^ CFU/g, for yeast and mold it is 100.0 CFU/g, and an absence of *E. coli* and *Salmonella* is required. During refrigerated storage, the lowest TPC and Y&M count were observed in Branded Meatballs followed by fish gelatin-incorporated meatballs. Control Meatballs had the highest TPC and Y&M count (*p* < 0.05) at 4 °C storage temperature compared to the branded and fish gelatin-incorporated meatballs. *E. coli* and *Salmonella* were not observed in any treatment groups, indicating hygienic preparation conditions, good quality ingredients, and the absence of any pathogenic microbes. From a consumer safety point of view, industries only supply meat products with a microbial load within an acceptable range [42]. On day three of storage in refrigerated conditions, the microbial load was beyond acceptable limits as prescribed by FSSAI. On day zero, during frozen conditions, no microbes were observed apart from the TPC and Y&M count. Furthermore, with an increase in storage duration, the overall growth of microorganisms decreased. This might be due to the reduced survival rate of microbes in frozen conditions [44]. Results from microbial studies suggested that meatballs stored at a refrigerated temperature were best consumed in the same day, and for long-term storage frozen temperatures are preferable. However, further processing and cooking using thermal methods such as frying can also alter the microbial load of the products. In addition, after cooking, it is advised to use good manufacturing practices (GMP) and good hygiene practices (GHP) to avoid cross-contamination.

## 4. Conclusions

In this study, the efficacy of pink perch gelatin as a fat replacer for the development of low-fat ready-to-cook chicken meatballs was evaluated on the basis of nutritional, technological, sensorial, and microbiological properties. The results suggested that the addition of fish gelatin improved the nutritional profile of the meatballs by reducing the fat content by 67.2% along with an increase of 20.1% in protein content in comparison to the control group. Fish gelatin addition also improved sensory properties and consumer acceptability of the product. At 5% fish gelatin concentration, the overall acceptability of the fish gelatin-incorporated meatballs was higher than the Branded Meatballs. The addition of fish gelatin provided good structuring and improved the textural properties of the meatball. The cooking, yield, and moisture retention of the meatballs significantly increased with the incorporation of fish gelatin. During storage at refrigerated and frozen conditions, fish gelatin improved the shelf-life of meatballs by effectively reducing oxidation due to its radical scavenging activity. The results also suggest that the addition of gelatin preserved the quality of the meatball by stabilizing pH and water holding capacity during storage. Overall, it can be concluded that the addition of fish gelatin can be used by meat industries to produce ready-to-cook chicken meatballs with better nutritional profiles and functional properties.

## Figures and Tables

**Table 1:** Composition of ingredients of the dough for ready-to-cook chicken meatballs *.

S. No.	Ingredient	Quantity (%)				
		Control	3FGMB	4FGMB	5FGMB	6FGMB
1	Chicken Meat	75.0	75.0	75.0	75.0	75.0
2	Hydrogenated vegetable oil	4.5	0.0	0.0	0.0	0.0
3	Pink perch Gelatin	0.0	3.0	4.0	5.0	6.0
4	Roasted Black Gram flour	8.0	8.0	8.0	8.0	8.0
5	Potato Starch	4.5	6.0	5.0	4.0	3.0
6	Spice Mix	2.7	2.7	2.7	2.7	2.7
7	Salt	1.8	1.8	1.8	1.8	1.8
8	Vegetables *	3.4	3.4	3.4	3.4	3.4

* 3FGMB: 3% Fish Gelatin Meatball, 4FGMB: 4% Fish Gelatin Meatball, 5FGMB: 5% Fish Gelatin Meatball, 6FGMB: 6% Fish Gelatin Meatball, Control: Control Meatball. Added in frozen form.

**Table 2:** Proximate composition of raw and fried meatballs *.

	Raw Meatballs						Fried Meatballs					
Parameter	Control	3FGMB	4FGMB	5FGMB	6FGMB	BMB	Control	3FGMB	4FGMB	5FGMB	6FGMB	BMB
Fat (%)	10.7 ± 0.9 ^c^	5.5 ± 1.7 ^de^	6.2 ± 1.1 ^de^	6.4 ± 1.2 ^d^	6.7 ± 0.4 ^d^	11.5 ± 0.9 ^c^	13.8 ± 0.9 ^b^	11.5 ± 0.5 ^f^	9.6 ± 1.1 ^ef^	7.7 ± 0.9 ^de^	6.9 ± 0.4 ^de^	15.6 ± 1.1 ^a^
Protein (%)	16.9 ± 0.0 ^i^	18.2 ± 0.0 ^h^	19.5 ± 0.0 ^g^	20.3 ± 0.0 ^f^	22.2 ± 0.0 ^e^	12.2 ± 0.0 ^j^	23.2 ± 0.0 ^d^	28.8 ± 0.0 ^c^	31.6 ± 0.0 ^b^	31.7 ± 0.0 ^b^	33.2 ± 0.0 ^a^	22.5 ± 1.3 ^e^
Moisture (%)	62.0 ± 0.6 ^c^	62.3 ± 0.0 ^bc^	63.2 ± 0.0 ^b^	64.5 ± 0.5 ^a^	65.0 ± 0.2 ^a^	62.2 ± 1.4 ^c^	51.8 ± 0.7 ^h^	53.3 ± 0.9 ^g^	54.4 ± 0.3 ^f^	55.8 ± 0.3 ^e^	56.7 ± 0.5 ^d^	53.3 ± 0.3 ^g^
Ash (%)	5.5 ± 0.2 ^a^	3.7 ± 0.1 ^bc^	3.9 ± 0.1 ^b^	3.5 ± 0.1 ^c^	3.8 ± 0.1 ^b^	3.7 ± 0.1 ^bc^	2.3 ± 0.1 ^ef^	2.3 ± 0.3 ^f^	2.4 ± 0.2 ^ef^	2.6 ± 0.1 ^e^	2.9 ± 0.1 ^d^	3.6 ± 0.4 ^bc^

* 3FGMB: 3% Fish Gelatin Meatball, 4FGMB: 4% Fish Gelatin Meatball, 5FGMB: 5% Fish Gelatin Meatball, 6FGMB: 6% Fish Gelatin Meatball, BMB: Branded Meatball, Control: Control Meatball. Values presented as mean value ± standard deviation; a–i (↓) different lowercase letters indicate significant difference between different meatballs (*p* < 0.05).

**Table 3:** Texture profile analysis of raw and fried meatballs *.

	Raw Meatball						Fried Meatball					
Parameter	Control	3FGMB	4FGMB	5FGMB	6FGMB	BMB	Control	3FGMB	4FGMB	5FGMB	6FGMB	BMB
Hardness (N)	16.6 ± 1.6 ^g^	21.3 ± 5.1 ^bc^	30.4 ± 6.5 ^f^	24.1 ± 3.2 ^fg^	27.6 ± 3.6 ^fg^	26.3 ± 5.7 ^fg^	189.3 ± 4.5 ^a^	166.0 ± 3.1 ^b^	156.0 ± 14.5 ^b^	139.5 ± 9.9 ^c^	125.6 ± 9.3 ^d^	108.1 ± 8.3 ^e^
Springiness (cm)	0.2 ± 0.1 ^d^	0.2 ± 0.1 ^d^	0.2 ± 0.0 ^d^	0.2 ± 0.0 ^d^	0.2 ± 0.0 ^d^	0.4 ± 0.1 ^c^	0.7 ± 0.0 ^ab^	0.7 ± 0.1 ^ab^	0.7 ± 0.1 ^ab^	0.7 ± 0.0 ^b^	0.7 ± 0.0 ^ab^	0.7 ± 0.0 ^a^
Cohesiveness	0.2 ± 0.0 ^d^	0.2 ± 0.0 ^d^	0.2 ± 0.0 ^d^	0.2 ± 0.0 ^d^	0.2 ± 0.0 ^d^	0.3 ± 0.1 ^bc^	0.3 ± 0.0 ^b^	0.3 ± 0.0 ^bc^	0.3 ± 0.1 ^b^	0.3 ± 0.0 ^c^	0.3 ± 0.0 ^bc^	0.4 ± 0.0 ^a^
Gumminess (N)	3.1 ± 0.4 ^d^	4.3 ± 1.0 ^d^	5.8 ± 1.5 ^d^	4.2 ± 0.6 ^d^	5.1 ± 0.9 ^d^	7.1 ± 2.5 ^d^	57.2 ± 1.6 ^a^	44.7 ± 1.4 ^b^	47.4 ± 10.4 ^b^	34.4 ± 1.6 ^c^	33.7 ± 4.1 ^c^	42.8 ± 4.9 ^b^
Chewiness (N.cm)	0.6 ± 0.2 ^d^	1.0 ± 0.5 ^d^	1.2 ± 0.3 ^d^	0.9 ± 0.1 ^d^	1.2 ± 0.4 ^d^	2.8 ± 0.9 ^d^	37.7 ± 0.6 ^a^	31.4 ± 9.8 ^b^	29.6 ± 3.0 ^b^	21.9 ± 1.4 ^c^	21.2 ± 1.5 ^c^	30.7 ± 3.5 ^b^

* 3FGMB: 3% Fish Gelatin Meatball, 4FGMB: 4% Fish Gelatin Meatball, 5FGMB: 5% Fish Gelatin Meatball, 6FGMB: 6% Fish Gelatin Meatball, BMB: Branded Meatball, Control: Control Meatball. Values presented as mean value ± standard deviation; a–g (↓) different lowercase letters indicate significant difference between different meatballs (*p* < 0.05).

**Table 4:** Cooking parameters of the fried meatballs *.

Parameters	Control	3FGMB	4FGMB	5FGMB	6FGMB	BMB
Yield (%)	70.9 ± 0.2 ^e^	75.6 ± 2.1 ^d^	78.8 ± 1.0 ^c^	81.8 ± 1.5 ^b^	85.6 ± 2.1 ^a^	83.8 ± 0.9 ^ab^
Shrinkage (%)	8.9 ± 0.2 ^a^	4.3 ± 0.1 ^bc^	4.2 ± 0.1 ^bc^	2.8 ± 1.2 ^c^	2.1 ± 0.1 ^c^	5.9 ± 2.7 ^b^
Moisture Retention (%)	44.0 ± 0.2 ^e^	47.8 ± 1.0 ^d^	49.8 ± 0.9 ^cd^	53.2 ± 0.5 ^b^	56.5 ± 0.9 ^a^	51.7 ± 1.2 ^bc^

* 3FGMB: 3% Fish Gelatin Meatball, 4FGMB: 4% Fish Gelatin Meatball, 5FGMB: 5% Fish Gelatin Meatball, 6FGMB: 6% Fish Gelatin Meatball, BMB: Branded Meatball, Control: Control Meatball. Values are presented as mean value ± standard deviation; a–e (↓) different lowercase letters indicate significant difference between different meatballs (*p* < 0.05).

**Table 5:** Color analysis of meatballs stored at 4 °C for 15 days *.

Days	Control			5FGMB			BMB		
	L *	A *	B *	L *	A *	B *	L *	A *	B *
0	53.87 ± 0.36 ^a^	14.50 ± 0.09 ^c^	28.83 ± 0.75 ^bc^	54.30 ± 1.39 ^a^	13.77 ± 0.03 ^b^	26.21 ± 1.84 ^a^	56.02 ± 0.06 ^a^	2.76 ± 0.03 ^c^	13.01 ± 0.04 ^a^
3	52.62 ± 0.53 ^b^	14.95 ± 0.13 ^c^	30.26 ± 0.46 ^a^	53.40 ± 0.09 ^ab^	13.87 ± 0.06 ^b^	28.47 ± 5.57 ^a^	55.34 ± 0.05 ^b^	3.03 ± 0.03 ^b^	13.08 ± 0.09 ^a^
6	52.47 ± 0.27 ^b^	15.52 ± 0.39 ^b^	28.48 ± 0.03 ^c^	53.15 ± 0.87 ^ab^	14.65 ± 0.62 ^a^	27.42 ± 1.36 ^a^	54.75 ± 1.29 ^c^	3.57 ± 0.14 ^b^	13.95 ± 0.88 ^a^
9	51.73 ± 0.65 ^b^	15.61 ± 0.19 ^b^	30.34 ± 0.87 ^a^	52.21 ± 0.08 ^ab^	14.92 ± 0.03 ^a^	26.83 ± 0.08 ^a^	53.85 ± 0.25 ^d^	3.78 ± 0.13 ^b^	12.96 ± 1.38 ^a^
12	50.79 ± 0.23 ^c^	15.96 ± 0.38 ^b^	29.65 ± 0.45 ^ab^	51.55 ± 0.63 ^bc^	15.04 ± 0.54 ^a^	28.68 ± 0.88 ^a^	53.76 ± 0.42 ^e^	4.14 ± 0.19 ^a^	13.07 ± 0.04 ^a^
15	50.43 ± 0.74 ^c^	16.64 ± 0.30 ^a^	28.31 ± 0.60 ^c^	50.75 ± 0.13 ^c^	15.19 ± 0.54 ^a^	28.33 ± 0.14 ^a^	52.46 ± 0.35 ^f^	4.63 ± 0.04 ^a^	12.66 ± 0.54 ^a^

* Control: Control Meatball, 5FGMB: 5% Fish Gelatin Meatball, BMB: Branded Meatball. Values presented as mean value ± standard deviation; a–d (↓) different lowercase letters indicate significant difference between different meatballs (*p* < 0.05).

**Table 6:** Color analysis of meatballs stored at −18 °C for 60 days *.

Days	Control			5FGMB			BMB		
	L *	A *	B *	L *	A *	B *	L *	A *	B *
0	53.87 ± 0.36 ^NS^	14.50 ± 0.09 ^c^	28.83 ± 0.75 ^b^	54.30 ± 1.39 ^NS^	13.77 ± 0.03 ^a^	26.21 ± 1.84 ^NS^	56.02 ± 0.06 ^a^	2.76 ± 0.03 ^d^	13.01 ± 0.04 ^NS^
15	53.62 ± 0.53 ^NS^	14.95 ± 0.13 ^bc^	30.26 ± 0.46 ^a^	54.20 ± 0.13 ^NS^	13.77 ± 0.21 ^a^	28.47 ± 5.57 ^NS^	54.34 ± 0.05 ^b^	3.63 ± 0.16 ^b^	13.08 ± 0.09 ^NS^
30	53.47 ± 0.27 ^NS^	15.52 ± 0.39 ^ab^	27.48 ± 0.03 ^c^	54.15 ± 0.87 ^NS^	13.65 ± 0.62 ^a^	27.42 ± 1.36 ^NS^	52.75 ± 1.29 ^c^	3.07 ± 0.04 ^c^	13.95 ± 0.88 ^NS^
45	53.73 ± 0.65 ^NS^	15.51 ± 0.19 ^ab^	30.34 ± 0.87 ^a^	54.21 ± 0.08 ^NS^	14.52 ± 0.31 ^b^	26.83 ± 0.08 ^NS^	52.85 ± 0.25 ^c^	3.48 ± 0.13 ^b^	12.96 ± 1.38 ^NS^
60	52.79 ± 0.23 ^NS^	15.66 ± 0.55 ^a^	29.65 ± 0.45 ^ab^	54.15 ± 0.72 ^NS^	14.94 ± 0.33 ^b^	26.68 ± 0.88 ^NS^	53.76 ± 0.42 ^bc^	3.84 ± 0.04 ^a^	13.07 ± 0.04 ^NS^

* Control: Control Meatball, 5FGMB: 5% Fish Gelatin Meatball, BMB: Branded Meatball. Values presented as mean value ± standard deviation; a–d (↓) different lowercase letters indicate significant difference between different meatballs (*p* < 0.05); NS indicated no significant difference between different meatballs.

**Table 7:** Total plate count, *Salmonella*, yeast and molds, and *E. coli* count (CFU/g) of meatballs stored at 4 °C for 15 days *.

Days	Total Plate Count (CFU/g)			*Salmonella* Count (CFU/g)			Yeast and Mold Count (CFU/g)			*E. coli* Count (CFU/g)		
	Control	5FGMB	BMB	Control	5FGMB	BMB	Control	5FGMB	BMB	Control	5FGMB	BMB
0	1.2 × 10^3^ ± 3.1 ^a^	8.9 × 10^2^ ± 3.0 ^b^	7.8 × 10^2^ ± 2.9 ^c^	ND	ND	ND	28.5 ± 1.5 ^a^	23.5 ± 1.4 ^b^	8.5 ± 0.9 ^c^	ND	ND	ND
3	TNTC	TNTC	TNTC	ND	ND	ND	TNTC	TNTC	TNTC	ND	ND	ND
6	TNTC	TNTC	TNTC	ND	ND	ND	TNTC	TNTC	TNTC	ND	ND	ND
9	TNTC	TNTC	TNTC	ND	ND	ND	TNTC	TNTC	TNTC	ND	ND	ND
12	TNTC	TNTC	TNTC	ND	ND	ND	TNTC	TNTC	TNTC	ND	ND	ND
15	TNTC	TNTC	TNTC	ND	ND	ND	TNTC	TNTC	TNTC	ND	ND	ND

* Control: Control Meatball, 5FGMB: 5% Fish Gelatin Meatball, BMB: Branded Meatball, TNTC: Too Numerous To Count. Values presented as mean value ± standard deviation; ND: Not Detected; a–c (↓) different lowercase letters indicate significant difference between different meatballs (*p* < 0.05).

**Table 8:** Total plate count, *Salmonella*, yeast and molds and *E. coli* count (CFU/g) of meatballs stored at −18 °C for 60 days *.

Days	Total Plate Count (CFU/g)			*Salmonella* Count (CFU/g)			Yeast and Mold Count (CFU/g)			*E. coli* Count (CFU/g)		
	Control	5FGMB	BMB	Control	5FGMB	BMB	Control	5FGMB	BMB	Control	5FGMB	BMB
0	1.2 × 10^3^ ± 3.1 ^a^	8.9 × 10^2^ ± 3.0 ^b^	7.8 × 10^2^ ± 2.9 ^c^	ND	ND	ND	28.5 ± 1.5 ^a^	23.5 ± 1.4 ^b^	8.5 ± 0.9 ^c^	ND	ND	ND
15	ND	ND	ND	ND	ND	ND	ND	ND	ND	ND	ND	ND
30	ND	ND	ND	ND	ND	ND	ND	ND	ND	ND	ND	ND
45	ND	ND	ND	ND	ND	ND	ND	ND	ND	ND	ND	ND
60	ND	ND	ND	ND	ND	ND	ND	ND	ND	ND	ND	ND

* Control: Control Meatball, 5FGMB: 5% Fish Gelatin Meatball, BMB: Branded Meatball. Values presented as mean value ± standard deviation; ND: Not Detected; a–c (↓) different lowercase letters indicate a significant difference between different meatballs (*p* < 0.05).

## Data Availability

The data presented in this study are available within the article.
